# Risk of Development of Resistance in Patients with Non-Cystic Fibrosis Bronchiectasis Treated with Inhaled Antibiotics

**DOI:** 10.1007/s13665-018-0202-7

**Published:** 2018-07-05

**Authors:** Kate H. Regan, Adam T. Hill

**Affiliations:** 1 The Queen’s Medical Research Institute, University of Edinburgh/MRC Centre for Inflammation Research, Edinburgh, UK; 2 Department of Respiratory Medicine, Royal Infirmary of Edinburgh, Edinburgh, UK

**Keywords:** Non-CF bronchiectasis, Inhaled antibiotics, Antibiotic resistance

## Abstract

**Purpose of Review:**

Bronchiectasis is a debilitating chronic lung disease characterised by recurrent bacterial infection and colonisation with significant associated morbidity and mortality. To date, there are few licenced treatments, and the mainstay of clinical management is prompt antibiotic therapy for exacerbations and regular airway clearance. Inhaled antibiotics are a potential long-term treatment for those with recurrent exacerbations, and represent an obvious advantage over other routes of administration as they achieve high concentrations at the site of infection whilst minimising systemic side effects. The main caveat to such treatment is the development of antimicrobial resistance due to altered selection pressures.

**Recent Findings:**

Numerous studies of various inhaled antimicrobials have demonstrated favourable safety and efficacy profiles for bronchiectasis patients with chronic infection, which are supportive of their use in clinical practice.

**Summary:**

There is no convincing evidence of treatment-emergent pathogens or pathogens developing resistance to the inhaled antibiotic therapy.

## Introduction

Bronchiectasis is a chronic lung disease characterised by permanent dilatation of the lower airways, resulting in a clinical syndrome of chronic productive cough and recurrent lower respiratory tract infections [1, 2]. Approximately 2–5 per 1000 population are affected by this debilitating lung disease, with incidence highest in patients aged > 60 [3, 4]. Bronchiectasis represents the common pathological endpoint of a variety of underlying conditions, including cystic fibrosis (CF). Bronchiectasis is generally categorised as either CF-related or non-CF-related bronchiectasis (NCFB), the latter of which will form the focus of this review. NCFB most frequently occurs following a severe lower respiratory tract infection or is idiopathic, though there are numerous potential aetiologies including allergic bronchopulmonary aspergillosis (ABPA), immune function disorders and ciliary dyskinesias [5]. The gold standard technique for the diagnosis of bronchiectasis is high resolution CT chest, which demonstrates bronchial dilatation to greater than the diameter of the accompanying pulmonary artery and a failure of small airways tapering [6•]. The resulting impairment in mucociliary clearance (MCC) leads to excess secretions in the lower airways, providing an ideal environment for bacterial infection to thrive [7].

In the early phases of the disease, infection is cleared by prolonged courses of oral or intravenous antibiotics. Over time, the cycle of recurrent infections progresses to bacterial colonisation in the majority of patients, leading to a progressive decline in lung function, worsening quality of life and increasing lung damage. Unlike in CF, where bacterial colonisation tends to follow a ‘classical’ pattern of early *Staphylococcus aureus* (SA) infection and almost inevitable later colonisation with *Pseudomonas aeruginosa* (PA), the chronic pathogens implicated NCFB are more heterogenous [8, 9]. The most common chronic infections in NCFB are *Haemophilus influenzae* (HI) and *Pseudomonas aeruginosa* (PA), with *Staphylococcus aureus* (SA), *Streptococcus pneumoniae* (SP), *Moraxella catarrhalis* (MC) and enteric gram-negative organisms also commonly cultured from lower respiratory tract secretions [10, 11]. In recent years, there has also been increasing focus on the role of non-tuberculous mycobacterial (NTM) infection, which is now known to affect up to 10% of the bronchiectasis population [12, 13]. Once established in the lower airway, bacteria become extremely difficult to eradicate from the lung.

Patients with bronchiectasis report poor health-related quality of life (HRQoL), significant fatigue and a high burden of respiratory symptoms, which are closely linked to bacterial load in the lower airway and the resultant respiratory and systemic inflammation [14–20]. Despite this, there remain few recommendations for their long-term management, with treatment focussed on rapid initiation of culture-targeted antibiotics for respiratory exacerbations and daily airway clearance. Given the role of bacterial infection in the severity of symptoms and disease progression, there has been much focus on the role of long-term antibiotic therapy in NCFB.

Inhaled antibiotics provide an obvious advantage over enterally or parenterally administered antibiotics, as they reach much higher concentrations at the site of infection whilst remaining at low levels in the systemic circulation, thereby reducing potential side effects of long-term use such as renal, hepatic or auditory dysfunction. Inhaled antibiotics have long been studied and licenced in the CF population, where long-term use of inhaled anti-pseudomonal antibiotics such as colistin and tobramycin have demonstrated excellent clinical efficacy in reducing lung function decline, frequency of exacerbations and delaying chronic infection [21–23]. Disappointingly to date, few high-quality trials of inhaled antibiotics in NCFB have been published, and no inhaled antimicrobial preparations are currently licenced for use in this patient group. Despite this, current guidelines recommend the off-label use of inhaled antibiotics in the management of patients with chronic lung infection, and use of nebulised colistin and gentamicin is common in clinical practice [6•, 24]. According to data from the most recent British Thoracic Society bronchiectasis audit, some 10% of British bronchiectasis patients were receiving long-term inhaled antimicrobial therapy, of whom 86% received colistin, 6% gentamicin, 4% tobramycin and 4% others [4].

One of the main, and understandable, concerns of using long-term inhaled antibiotics as a suppressive therapy in both CF and NCFB is the emergence of resistant bacterial strains, further complicating the management of an already extremely challenging group of patients. Traditionally, we have assumed that the chronic exposure of a pathogen to a level of a specific antimicrobial that is insufficient to eradicate that pathogen will lead to the development of resistant strains via altered selection pressures. However, it is difficult to know whether established minimum inhibitory concentrations (MICs), developed in conjunction with parenteral break points, are applicable when the airway concentration of inhaled antibiotics significantly exceeds that which can be safely be achieved via the parenteral route due to the risk of systemic toxicity [25–27]. Furthermore, the MIC standards used by studies vary according to their geographical location, with most European centres ascribing to EUCAST standards, and those in the USA utilising the CLSI breakpoints [28, 29].

To date, most inhaled antibiotic studies have focused on patients colonised with PA, likely since numerous studies have independently linked this with increased morbidity and mortality, and because its management has been so extensively studied in CF [17, 30–32]. Chronic PA infection affects approximately 20% of NCFB patients, but the role of colonisation with other pathogens in disease course is less well defined [33, 34]. In this review article, we will focus on the risk of developing resistance in those taking long-term inhaled antibiotic therapy for NCFB.

## Tobramycin

### Couch [35]

This randomised placebo-controlled trial used tobramycin solution for inhalation (TSI) (TOBI®) for 4 weeks in patients colonised with PA. Seventy-four patients were enrolled and were equally divided between the treatment (300 mg TSI BD for 28 days) and placebo (1.25 mg quinine sulphate) arms, with the primary outcome being a reduction in sputum PA density. The study demonstrated a significant reduction in sputum bacterial load in the treatment group, with a mean reduction in PA bacterial load of 4.8 log_10_ and 4.54 log_10_ at 2 and 4 weeks respectively. Some regrowth was noted 2 weeks following discontinuation of therapy. At enrolment, all patients had tobramycin sensitive PA. 3/36 (8%) treated and 1/34 (3%) placebo patients developed PA isolates with tobramycin MICs ≥ 16 μg/mL (tobramycin parenteral breakpoint) during the study. The duration of follow-up was insufficient to determine whether these strains remained present following study discontinuation. There was no discussion of emergence of opportunistic bacterial species, though the effect of treatment on co-colonising pathogens was reviewed. Resistance was not tested for these groups.

### Scheinberg et al. [36]

This open-label, uncontrolled study of 300 mg BD of TSI consisted of 3x 2-week on/off treatment cycles and a 40-week follow-up period via review of medical notes. Forty-one patients with severe bronchiectasis colonised with PA were enrolled in this trial, whose primary outcome measure was improvement HRQoL. They demonstrated a mean reduction of 1.5 units in mean pulmonary total symptom severity score and 9.8 units in the St. George’s respiratory questionnaire (SGRQ) [37]. They reported an eradication rate of 22.2%; quantitative sputum bacteriology was not reported. Two (5%) of the study participants developed PA isolates with tobramycin MICs ≥ 16 μg/mL (16 and 128 μg/mL) at the end of treatment. No further resistance data following the end of the treatment period is reported.

### Orriols et al. 1999 [38]

In this early study, Orriols et al. conducted an open-label 12-month trial of nebulised ceftazidime 1000 mg BD and tobramycin 100 mg BD vs. standard care. They recruited patients with chronic PA infection who had a 2-year history of frequent exacerbations and difficult to control symptoms. Following a 2-week course of intravenous ceftazidime and tobramycin, patients were randomly allocated to receive treatment (*n* = 7) or standard care (*n* = 8). During the course of the trial, patients admitted with an exacerbation who subsequently had sputum cultures that demonstrated resistance were converted to piperacillin (100 mg BD) in place of ceftazidime and amikacin (100 mg BD) in place of tobramycin for the remainder of the study. The primary outcome measure was the number and duration of hospital admissions. Patients in the treatment arm had significantly fewer admissions; mean (± SEM) 0.6 (± 1.5) vs. 2.5 (± 2.1) and a shorter duration of admission, 13.1 (± 34.8) days and 57.9 (± 41.8) days respectively than those in the standard care group. There was no evidence of emergence of new opportunistic pathogens within either group and no successful eradication, though two treated and one untreated patient had transiently negative cultures. Of the treated group, one patient developed a tobramycin-resistant PA isolate without clinical worsening. In the group receiving standard care, four patients developed in vitro resistance to ceftazidime, one of whom also isolated tobramycin-resistant PA necessitating a change in antibiotic treatment. Overall, the incidence of resistant isolates was therefore higher in the group not receiving inhaled antibiotics, who had more admissions and thus more exposure to intravenous antibiotics.

### Barker et al. [39]

Barker and colleagues conducted a 4-week double-blinded RCT of 300 mg BD of nebulised TSI (TOBI®) vs. placebo (1.25 mg quinine sulphate), who were reviewed at 0, 2, 4 and 6 weeks (2 weeks post completion of treatment). Clinically stable patients with a history of chronic PA infection who could produce a sputum sample with at least 10^4^ CFU/g PA were recruited. The primary outcome measure was a reduction in sputum PA bacterial density. They demonstrated a significant reduction in sputum bacterial load at all time points; at week 4, the reduction was 4.54 log_10_ CFU/g vs. no change in the placebo group. At week 6, eradication was achieved in 35% of treated vs. 0% of placebo. Twenty-six percent of treated and 14% of the placebo group had a PA isolate at week 6 that had ≥ 4-fold increase in MIC vs. baseline. However, only 11% treated vs. 3% placebo had isolates with a MIC high enough to be considered resistant (≥ 16 μg/mL) [29]. There was no discussion of treatment-emergent pathogens.

### Drobnic et al. [40]

Drobnic et al. recruited patients chronically colonised with PA. This double blind, placebo-controlled crossover trial consisted of two arms of twice-daily nebulised tobramycin 300 mg or 0.9% NaCl (placebo) for 6 months, with a 1 month washout period between. Thirty patients chronically colonised with PA received 2 weeks of intravenous ceftazidime and tobramycin to achieve clinical stability prior to the initiation of nebulised therapy. Patients with PA isolates resistant to tobramycin (zone diameter ≥ 8 mm with a 0.01 mm tobramycin disk) were excluded from the trial. Primary endpoints were the number and duration of hospital admissions. Secondary endpoints included pulmonary function, days of antibiotic treatment, quality of life, sputum bacterial load, evidence of resistant strains and emergence of opportunistic bacteria. The trial demonstrated a significant reduction in the number mean, ± SD 0.15 ± 0.37 vs. 0.75 ± 1.16 and duration of admissions, 2.05 ± 5.03 days vs. 12.65 ± 21.8 days in the tobramycin and placebo arms respectively. There was also a non-significant trend towards decreased number of days of antibiotic therapy. There was no evidence of effect on pulmonary function, quality of life or frequency of exacerbations. Emergence of tobramycin-resistant PA was seen in two patients during the treatment arm and two patients during the second arm of placebo treatment. Two months following the end of the treatment period, all patients had fully susceptible PA (MIC < 8 μg/mL) in their sputum. Serial cultures demonstrated the emergence of *Alcaligenes xylosoxidans* and *S. pneumoniae* in two patients during the treatment arm and *Acinetobacter* species and *Stenotrophomonas maltophilia* during the placebo arm in two others. Overall, there was no difference in the emergence of bacterial resistance or acquisition of new opportunistic pathogens in response to treatment over the study duration.

### Orriols et al. 2015 [41]

Similarly to the above trials, Orriols et al. conducted a double-blinded RCT of 300 mg BD nebulised TSI (TOBI®) over 3 months in patients with first isolation of PA in sputum. Experimental treatment was initiated following a 2-week course of intravenous ceftazidime and tobramycin within 4 weeks of PA detection. Due to the high rates of tobramycin-associated bronchospasm reported in numerous previous trials of both CF and NCFB, patients also received a short-acting bronchodilator 1 h prior to treatment. The primary outcome measure was the proportion of PA-free patients; at the end of month 1, 90.9% of treated vs. 76.5% of placebo had negative sputum cultures, compared with 54.5 and 29.4% respectively at the end of the study. In addition, as found by Drobnic et al., the trial demonstrated a significant reduction in the number and length of admissions, and additionally reported a significant increase in the number of patients with PA negative cultures at the end of the study period. There was no improvement in pulmonary function tests, consistent with other trials, and no evidence of systemic toxicity. No tobramycin-resistant isolates of PA were detected at any time point during the course of the study. Opportunistic pathogens, including *S*. *maltophilia* and *A*. *xylosoxidans*, were detected in two treated and six placebo patients, suggesting no trend towards treatment-emergent pathogens.

## Ciprofloxacin

### ORBIT-2 [42]

ORBIT-2 was a multi-centre RCT examining the effect of a twice-daily liposomal ciprofloxacin DPI vs. placebo over a 6-month period in 28-day on/off cycles. They recruited 42 subjects with a history of chronic ciprofloxacin-sensitive PA infection with at least 2 exacerbations in the preceding year. The primary outcome measure was a reduction in sputum bacterial load at the end of the first 28-day treatment cycle, with mean (SD) 4.2 (3.7) vs. − 0.08 (3.8) log_10_ CFU/g reduction in treated vs. placebo. This persisted to 84 days post treatment initiation, and 60 vs. 14% had successful PA eradication at 28 days. They also demonstrated a significant increase in time to first pulmonary exacerbation, though there was no significant effect on HRQoL or pulmonary function. Emergence of new opportunistic sputum pathogens was seen in 55% of placebo and 45% of treated patients, with the most common new isolate being *S*. *maltophilia*. Ciprofloxacin resistance was determined according to CLSI breakpoints; 38% of placebo patients isolated an intermediately sensitive or resistant PA isolate at any time point vs. 50% of treated subjects. The change in MIC of the most resistant isolates identified at 28 days did not differ significantly between the two groups.

### Wilson et al. [43]

This phase II multi-centre RCT explored the use of BD ciprofloxacin 32.5 mg DPI over 4 weeks in the management of adult bronchiectasis patients with chronic airway pathogens who were frequent exacerbators (≥ 2 per year). Subjects who cultured PA, SA, SP, HI, MC, *S. maltophilia*, *Achromobacter xylosoxidans* or *Enterobacteriaceae* were eligible for study entry. The primary endpoint was a reduction in sputum bacterial load at the end of treatment (day 28), and the mean (range) reductions were 3.62 log_10_ (9.78–5.02) CFU/g in the active arm vs. 0.27 (7.95–5.25) log_10_ CFU/g in the placebo arm. There was also a non-significant trend towards reduced bacterial load at follow-up days 42 and 56 before a return to baseline at the end of study day 84. At day 8, pathogen eradication was achieved in 48% treated vs. 12% of the placebo group. Twelve treated and 24 placebo patients had sputum cultures positive for alternative respiratory pathogens vs. baseline; *S*. *maltophilia* was the most frequent new isolate. Increases in bacterial MIC to > 4 mg/L (ciprofloxacin parenteral break point) were reported in six treated and zero placebo patients; of these isolates only one still had an elevated MIC at the end of the study.

### RESPIRE I and II [44•, 45•]

The RESPIRE trials were multi-centre double-blinded RCTs conducted over 48 weeks; consisting of treatment with ciprofloxacin DPI 32.5 mg BD in either 14- or 28-day on/off cycles vs. placebo. Recruited patients had idiopathic or post-infective bronchiectasis and a history of recurrent exacerbations. All were chronically colonised with PA, HI, MC, SA, SP, SM or *Burkholderia cepacia* complex at baseline. Each trial recruited over 400 patients with high completion rates and study drug compliance > 90% in all groups. RESPIRE I demonstrated that ciprofloxacin DPI administered in 14-day on/off cycles significantly reduced the exacerbation rate by 39% over 48 weeks (mean number of exacerbations 0.6 vs. 1.0 treatment vs. pooled placebo), along with a significant increase in pathogen eradication and improvement in HRQoL as assessed by the SGRQ. The outcomes for the 28-day on/off cycles were not statistically significant. In RESPIRE II, there was a minor trend towards prolonged time to the next exacerbation but the overall exacerbation rate in participants was low, which was believed to play a role in the lack of significant evidence of treatment effect. The RESPIRE I trial reported that 24.5% of their patients demonstrated an elevated ciprofloxacin MIC at baseline. Interestingly, subgroup analyses did not demonstrate reduced efficacy in these patients, further supporting the hypothesis that the higher antimicrobial concentrations achieved in the lung following the use of inhaled antibiotics renders MIC data based on parenteral breakpoints irrelevant. Across the course of the study, 20.4% of the 14-day, 9.2% of the 28-day and 12.3% of the pooled placebo arms demonstrated an elevated MIC (relative to the CLSI breakpoint standards) in at least one isolate at any time point. At the end of the study visit (8 weeks post treatment cessation), 6.3% of participants had newly resistant isolates—7.3, 9.2 and 2.2% of the 14, 28 and placebo arms respectively. Results from RESPIRE II were similar. The authors postulate that any treatment-related MIC increase is likely to be offset by the overall decrease in bacterial load, reduced exacerbation rate and consequent reduction in systemic antimicrobial therapy.

## Colistin

### Haworth et al. [46]

Despite the fact that colistin is the most frequently used off-label inhaled antibiotic in the management of chronic bronchial infection in NCFB, the trial by Haworth et al. is the only double-blinded RCT examining its role in this population. The bulk of the evidence supporting its use in the management of PA colonisation is extrapolated from the CF world, where it has been a mainstay of treatment for several decades. Haworth’s group conducted a trial of 6 months duration exploring the use of 1 million IU BD of nebulised colistin vs. placebo (0.45% NaCl) in adult bronchiectasis patients with a history of chronic PA infection following a course of anti-pseudomonal antibiotics for management of a pulmonary exacerbation. Their primary endpoint was time to the first exacerbation, and time to the first exacerbation based on adherence to study drug was amongst their secondary end points. Forty-nine percent of patients in the colistin group vs. 59% in the placebo group experienced an exacerbation, with median time to exacerbation 165 vs. 111 days respectively, which was not statistically significant. However, patients with a compliance of ≥ 80% to study medication had a significant increase in time to first exacerbation, with 35% of treated and 82% of placebo patients experiencing an exacerbation; median time to the first exacerbation could not be calculated in the colistin group due to the number of patients completing the treatment course. They also demonstrated improved HRQoL (SGRQ) with no effect on pulmonary function. No colistin-resistant PA isolates (as per EUCAST guidelines) were identified at any time point during the study and there was no difference between the emergent new pathogens between the treated and untreated groups.

## Gentamicin

### Murray et al. [47]

Following colistin, nebulised gentamicin is the next most frequently used off-label nebulised antibiotic in the management of NCFB complicated by chronic bronchial infection. The rationale behind the use of gentamicin is its broad spectrum of action, but its use intravenously confers a cumulative risk of nephro- and ototoxicity. In this single-blinded RCT, NCFB patients with chronic lung infection with any potential respiratory pathogen and frequent (≥ 2/year) exacerbations were randomised to receive 80 mg BD of nebulised gentamicin or 0.9% NaCl over 12 months and were reviewed at 3-monthly intervals. The primary end points were sputum bacterial density and pathogen eradication. The trial reported a significant reduction in sputum bacterial load at 12 months in the gentamicin-treated group, with median bacterial density (range) 2.96 (1.0–5.9) log_10_ CFU/mL in the gentamicin treated group vs. 7.67 (7.34–8.17) log_10_ CFU/mL in the placebo group. 30.8% of PA-colonised patients and 92.8% of those colonised with other pathogens had negative sputum cultures at the end of treatment with gentamicin. In addition, the treatment group reported significantly increased exercise tolerance, HRQoL (SGRQ), fewer exacerbations, increased time to first exacerbation and decreased sputum inflammatory indices during treatment. At 3-month post treatment, cessation indices had returned to baseline, highlighting that continuous treatment is likely to be required for sustained clinical improvement. As with other trials, there was no effect demonstrated on pulmonary function, and no evidence of systemic toxicity. No gentamicin-resistant or intermediate resistant isolates of PA or enteric gram-negative organisms were isolated at baseline, end of treatment or end of study. Other pathogens were not tested for resistance. Furthermore, there was no evidence of new treatment-emergent pathogens.

## Aztreonam

### AIR-BX1 and 2 [48]

The AIR-BX trials were identical multi-centre randomised controlled trials of aztreonam lysine solution for inhalation (AZLI), an inhaled anti-pseudomonal antibiotic with a strong evidence base in the management of chronic airway infection in the CF population [49–51]. Patients chronically colonised with gram-negative organisms, (other than *H*. *influenzae* alone), and a history of chronic sputum production were recruited to receive two cycles of 4 weeks of nebulised AZLI 75 mg TDS or matched placebo followed by 4 weeks off treatment. The primary endpoint was change from baseline in quality of life bronchiectasis respiratory symptoms score (QOL-B-RSS) at 4 weeks [52, 53]. In AIR-BX1, 134 were randomised to AZLI and 132 to placebo; in AIR-BX2, 136 received AZLI and 138 placebo. The mean adherence to study medication was high in both trials across treatment allocations. In AIR-BX1, following 4 weeks of treatment, there was a mean (SE) increase in QOL-B-RSS of 6.4 (1.4) points in the AZLI group vs. 5.6 (1.4) points in the placebo group. Similarly at 12 weeks, the mean (SE) increase was 5.7 (1.6) points vs. 4.4 (1.5) points for the AZLI and placebo groups respectively, with neither time point demonstrating statistical significance. In AIR-BX2, the mean (SE) increase was 7.9 (1.3) points for the AZLI and 3.3 (1.3) points for the placebo groups at 4 weeks, and 5.3 (1.4) points vs. 4.1 (1.4) points for the AZLI and placebo groups at 12 weeks. At 4 weeks, the mean increase between the AZLI and placebo groups of 4.6 points was statistically significant (*p* = 0.011); however, the minimum clinically important difference validated for QOL-B is 8 points, and as such, this result was not deemed to be clinically relevant [54]. The proportion of patients with detectable organisms in sputum was reduced in the AZLI-treated groups after baseline, with AZLI also demonstrating decreased sputum bacterial load. In AIR-BX1, 15% of AZLI-treated vs. 6% of placebo-treated patients with detectable organisms in sputum at 4 weeks demonstrated increases in aztreonam MIC of 4-fold or greater. Similarly at 12 and 16 weeks respectively, 35 and 23% of AZLI and 11 and 14% of placebo isolates demonstrated MIC increase of ≥ 4-fold. In AIR-BX2, at 4, 12 and 16 weeks respectively, increases in MIC of ≥ 4-fold were seen in 23, 34 and 20% of those receiving AZLI vs. 7, 11 and 6% of the placebo group. Actual MIC values for the organisms were not reported in the study; therefore, it is difficult to determine whether these increases correspond with clinically significant resistance. There was no discussion of treatment-emergent pathogens.

## Discussion

Aside from the tolerability of inhaled antibiotics, the main clinical concern with their long-term use in chronic bronchial infection is the development of resistant bacterial strains. There has been much media focus on the so-called antibiotic apocalypse and the imminent arrival of the ‘post-antibiotic era’. Given the challenges modern healthcare faces in the form of MDR infections such as MRSA, vancomycin-resistant enterococci and *Clostridium difficile* infection, which have arisen largely due to historical over-prescription of antibiotic therapy, it is perhaps unsurprising that such concerns are at the forefront of most clinician’s minds. Furthermore, the CF and NCFB populations are unique in their pathophysiology with respect to the fact that these infections may need suppressive therapy over several decades; which has been hitherto uncharted territory in terms of antibiotic prescribing. Nonetheless, observational studies and clinical trials have repeatedly demonstrated the correlation between worse clinical outcomes and bacterial load, and as such suppressive treatment of these chronic infections may be beneficial in the management of these patients for whom eradication is unlikely to be a realistic clinical goal [19, 20].

Above, we have reviewed numerous studies examining the role of inhaled antibiotic preparations in the management of NCFB, and it is clear that most studies are supportive of their role in reducing sputum bacterial load with consequent improvement in exacerbation rates, frequency and duration of hospital admissions and HRQoL [35, 36, 38–43, 44•, 45•, 46–48]. See Table 1 for a summary of findings. Reassuringly, the studies exploring the use of tobramycin, colistin and gentamicin did not demonstrate significant emergence of antimicrobial-resistant isolates in sputum and any transient increases in MIC generally returned to baseline following treatment cessation. In addition, there was little evidence that reduction in bacterial load or apparent eradication of the dominant pathogen was associated with treatment-emergent pathogens.

**Table 1:** Summary of resistance and treatment-emergent pathogen data from included studies

Study	Active arm	Placebo arm	Study type	Study length	Drug resistance	Treatment-emergent pathogens
Couch [35]	Tobramycin 300 mg BD	Quinine sulphate 1.25 mg BD	RCT	4 weeks	3/36 (8%) tobramycin and 1/34 (3%) placebo developed resistant isolates (not significantly different).	Not reported
Scheinberg et al. [36]	Tobramycin 300 mg BD	–	Open-label, uncontrolled	12 weeks in 2-week on/off cycles	2/41 (5%) patients developed resistant isolates at the end of treatment.	Not reported
Orriols et al. 1999 [38]	Ceftazidime 1000 mg BD AND Tobramycin 100 mg BD	Standard care	Open-label	52 weeks	1/7 (14%) treated had a tobramycin-resistant isolate. 4/8 (50%) standard care had ceftazidime-resistant isolates, of which 1 was also tobramycin resistant.	No treatment-emergent pathogens
Barker et al. [39]	Tobramycin 300 mg BD	Quinine sulphate 1.25 mg BD	Double-blinded RCT	4 weeks	11% treated and 3% placebo developed resistant PA isolates at the end of treatment.	Not reported
Drobnic et al. [40]	Tobramycin 300 mg BD	0.9% NaCl 8 mL BD	Double-blinded cross over RCT	2 × 26-week cycles with 4-week washout	2 tobramycin and 2 placebo-treated patients had resistant isolates mid-study. At EOS, no resistant isolates were identified.	2 treated patients cultured *A. xylosoxidans* and *S. pneumoniae.* 2 placebo patients cultured *S. maltophilia*. (No significant difference)
Orriols et al. 2015 [41]	Tobramycin 300 mg BD	0.9% NaCl	Double-blinded RCT	12 weeks	No tobramycin-resistant isolates were detected at any time point.	No significant difference
ORBIT-2 (42)	Liposomal ciprofloxacin 150 mg and free ciprofloxacin 60 mg BD	Control liposomes 15 mg and 0.9% NaCl	RCT	26 weeks in 4-week on/off cycles	Day 28: median (range) change in PA MIC from baseline was 0 (− 0.5 to + 31) vs. 0 (− 0.75 to + 0.5) in treated vs. placebo groups. (Not significant). EOS: no significant difference between groups.	2 treated and 6 untreated patients cultured *S. maltophilia* or *A. xylosoxidans*. (No significant difference)
Wilson et al. [43]	Ciprofloxacin 32.5 mg BD	Matched placebo DPI	RCT	4 weeks	6 ciprofloxacin and 1 placebo patients had a resistant isolate at any time point. At EOS, only 1 isolate from the treated group maintained resistance.	12 ciprofloxacin-treated and 24 placebo patients cultured new opportunistic pathogens. The most common isolate was *S. maltophilia*.
RESPIRE I and II [44•, 45•]	Ciprofloxacin 32.5 mg BD	Matched placebo DPI	Double-blinded RCT	48 weeks in EITHER 2-week OR 4-week on/off cycles	At the EOS 6.3% of participants had newly resistant isolates vs. baseline, 7.3% in the 14-day; 9.2% in the 28-day and 2.2% in the pooled placebo groups.	Not reported
Haworth et al. [46]	Colistin 1 million IU BD	0.45% NaCl	Double-blinded RCT	26 weeks	No colistin resistant isolates were detected at any time point.	No changes in pathogen isolation frequency from baseline to EOS.
Murray et al. [47]	Gentamicin 80 mg BD	0.9% NaCl	Single-blinded RCT	52 weeks	No gentamicin resistant PA isolates were detected at any time point. Other pathogens were not tested.	No increase in treatment-emergent pathogens.
AIR-BX1 [48]	Aztreonam Lysine 75 mg TDS	Matched placebo	Double-blinded RCT	16 weeks with 2 × 4-week on/off cycles	4 weeks, 15% AZLI and 6% placebo ≥ 4-fold increased MIC. 12 weeks, 35% AZLI and 11% placebo ≥ 4-fold increased MIC. 16 weeks, 23% AZLI and 14% placebo ≥ 4-fold increase in MIC.	No discussion of treatment-emergent pathogens.
AIR-BX2 [48]	Aztreonam Lysine 75 mg TDS	Matched placebo	Double-blinded RCT	16 weeks with 2 × 4-week on/off cycles	4 weeks, 23% AZLI and 7% placebo ≥ 4-fold increased MIC. 12 weeks, 34% AZLI and 11% placebo ≥ 4-fold increased MIC. 16 weeks, 20% AZLI and 6% placebo ≥ 4-fold increase in MIC.	No discussion of treatment-emergent pathogens.

*RCT*, randomised controlled trial; *PA*, *Pseudomonas aeruginosa*; *EOS*, end of study; *DPI*, dry powder inhaler; *AZLI*, aztreonam lysine solution for inhalation; *MIC*, Minimum Inhibitory Concentration

The reasons for this lack of resistance development are likely to be multifactorial and are yet to be fully explored. It seems likely that traditional MIC as determined in conjunction with parenteral breakpoints are unlikely to be applicable in the case of inhaled antibiotics, which lead to sputum concentrations significantly greater than that which can be safely administered parenterally without toxic effects. As the use of inhaled antibiotics is further explored, airway-adjusted MICs may be developed that correspond more directly with the likely concentrations achieved via the inhalational route. It is possible that any transient and potentially clinically insignificant increases in MIC will be offset by the benefits of reduced exacerbations and thereby exposure to systemic antimicrobial therapies.

In those trials examining the role of ciprofloxacin and aztreonam, the data regarding increases in resistance were less clear cut. Both the RESPIRE and AIR-BX trials demonstrated that a higher proportion of patients receiving active treatment developed isolates with significantly increased MIC than those receiving placebo at the end of treatment visits. In addition, even following a period off treatment, the percentage of patients with resistant isolates was still increased compared to baseline in the treatment vs. the placebo groups, though to a lesser extent than isolates taken immediately following active treatment. Interestingly, the RESPIRE trials included a sub-analysis of those patients who started the trial with ciprofloxacin-resistant PA isolates, which demonstrated no difference in treatment efficacy for these patients, underpinning the view that in vitro resistance does not necessarily transfer to lack of in vivo drug efficacy. In vitro resistance, in addition, may not be applicable as inhaled antibiotics achieve concentrations several folds higher than the MIC. Furthermore, it would be interesting to explore the relationship between these increases in MIC and the intermittent modality of drug administration utilised in these particular trials. Historically, 28-day on/off treatment cycles have been favoured in the CF population as they are thought to reduce development of bacterial resistance. However, several of the studies we have discussed above highlight the necessity of continuous therapy in maintaining clinical efficacy in the NCFB population, and perhaps this approach is required to ensure continued bacterial suppression and reduce the development of resistant isolates in NCFB.

The majority of the trials we have discussed relate only to chronic PA bronchial infection, which whilst a significant complication of late-stage NCFB is not the most common colonising airway pathogen. It is true that unlike with PA, the effect of colonisation with other potential respiratory pathogens is perhaps less definitively linked with increased morbidity and mortality. Nonetheless, development of safe and effective inhaled treatments that can target the wide range of infecting pathogens seen in NCFB may be a useful therapeutic strategy. Few of the trials enrolling such patients have comprehensively reported MIC testing across all pathogens, but the data available are suggestive of low rates of resistant isolates.

None of the trials we have identified have explored the use of inhaled antibiotic preparations in NCFB for periods > 1 year, which given the chronic nature of bronchial infection in these patients and the fact that all trials indicate the need for continuous therapy to achieve sustained clinical improvement is perhaps the biggest caveat to these conclusions. It is certainly clear that observational studies of patients on inhaled antimicrobial therapy will need to be extended over many years to truly determine their long-term impact. Furthermore, the role of intermittent vs. continuous treatment with the same inhaled antimicrobial has not been examined in any of the trials we have discussed. Exploration of this is particularly important in order to optimise therapeutic regimens to be safe, tolerable and efficacious in NCFB, whilst minimising the emergence of resistant bacterial strains.

In our opinion, the current evidence base collectively leans in favour of the benefits of inhaled antimicrobial therapy in improving patient morbidity and HRQoL, with little evidence of emerging bacterial resistance, treatment-emergent pathogens or systemic toxicity up to 1 year of therapy.
